# Supplementary material for: Deposition of ultra-thin coatings by a nature-inspired Spray-on-Screen technology
Source: Commun Eng. 2023 Jun 22;2:42. doi: 10.1038/s44172-023-00093-0 (PMC10955976; doi:10.1038/s44172-023-00093-0)
Supplement: Supplementary file 2 — Supplementary Information [file 44172_2023_93_MOESM2_ESM.pdf]

Supplementary information for

Deposition of ultra-thin coatings by a nature-inspired Spray-on-Screen technology

Rachith Shanivarasanthe Nithayananda Kumar[1,2], Andrea Valencia Ramirez[1,2], Pieter Verding[1,2], Philippe Nivelle[1,2], Prof. Frank Renner[1,2], Prof. Jan D'Haen[1,2], Prof. Wim Deferme[1,2]**

[1]Hasselt University, Institute for Materials Research (IMO-IMOMEC); 3590 Diepenbeek, Belgium

[2]IMEC vzw, Division IMOMEC; Wetenschapspark 1, B-3590 Diepenbeek, Belgium

*rachith.shanivarasanthe@uhasselt.be

*wim.deferme@uhasselt.be

Supplementary Note 1

Ultrasonic spray coating(USSC) is a R2R compatible technique, which can deposit a uniform thin coating[1] on any substrate with high accuracy, good resolution and very high transfer rate. However, achieving these homogeneous layers for thicknesses below 15nm on large areas possesses extreme difficulties due to the nature of the droplet dynamics. Ultrasonic spray coating utilizes the vibrations produced by the mechanical expansion and contraction of piezoelectric transducers inside the nozzle to atomize the droplet[2–4]. The atomization produces droplets in the range of 20 - 90µm[5], which are forced to impinge on the substrate by using a carrier gas (often nitrogen). Here, amongst many properties, the surface tension of the liquid and the surface energy of the substrate play a key role in the formation of an ultra-thin coating. Droplet size and shape when the droplet touches the substrate, determine the regime of wetting. For a very small droplet such as is the case for ultrasonic spray coating the size of which is less than the capillary length $l_c=(\rho g/\gamma)^{1/2}$, (where ρ is the density, γ is the surface tension of the liquid), the effect of surface tension is dominant over gravity. Therefore, the shape of the droplet is found to be a spherical cap for USSC.

Figure 1 dipicts the regimes of different solution processing techniques in relationship with the scalability and minimal layer thickness as described in the literature.

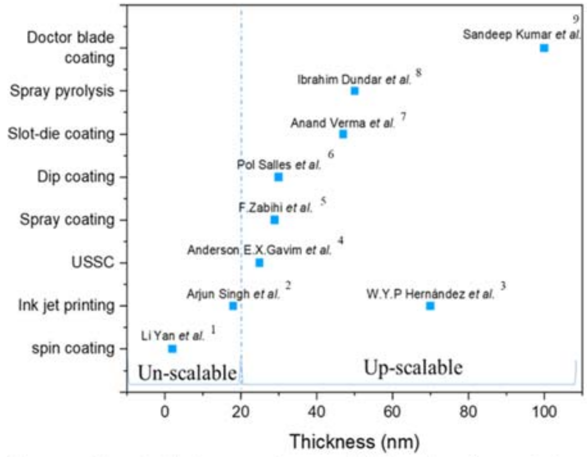

Figure 1. Minimum achieved thickness and up-scalability of various solution processing techniques

Supplementary Note-2

The spray on screen technology is inspired by the hydrophobic behavior of the water striders, which also induces dynamic wetting is shown in the **Figure S2**

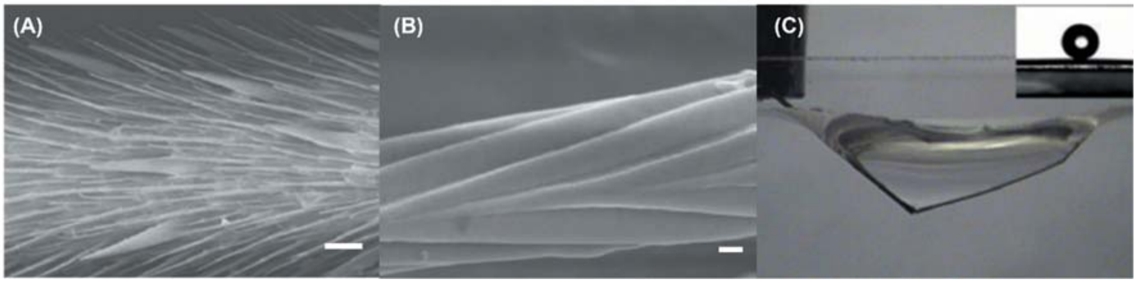

Figure 2. (**A**) Scanning electron microscope images of a leg showing numerous oriented spindly macrosetae (**B**) and the fine nanoscale grooved structures on a seta (**C**) Typical side view of a maximal-depth dimple (4.38 ± 0.02 mm) just before the leg pierces the water surface. Inset, a water droplet on a leg; this makes a contact angle of 167.6 ±4.4°. Scale bar (A) 20 µm: (B),200nm (reproduced with permission from Springer Nature) [6]

Supplementary Note-3: Measurement of the water contact angle on the screen

To measure the behavior of the water droplet on the screen mesh, contact angle measurements were done and as can be seen from **Figure 3**, the screen mesh has a water contact angle of ~104 degrees, which signifies that the screen is hydrophobic in nature. The image was taken on the same mesh screen (**Figure 2 main article**) with micro-droplets showing the conversion of microdroplets into milli-droplets with an equilibrium contact angle, these droplets are later used to create the thin liquid film over the substrate of interest.

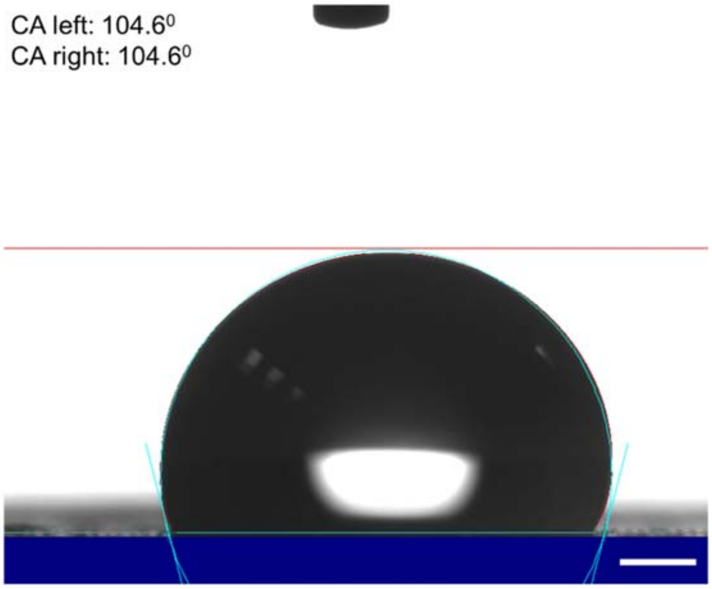

Figure 3. Water contact angle on the screen mesh (Scale 0.5 mm)

Supplementary Note-4: Conversion of microdroplets into milli droplets on the screen mesh

The microdroplets from the USSC are made to imping on the screen mesh, and irrespective of the surface tension of the formulation being used, the micro droplets get converted in to milli droplets on the screen mesh

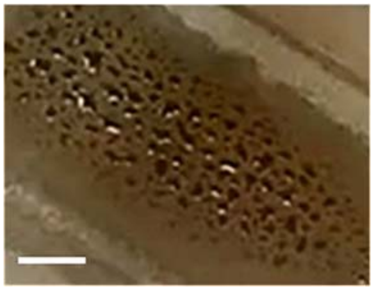

Figure 4. Formation of milli droplets on the mesh upon using water as a solvent to deposit silver nanoparticles (Scale 1cm)

Supplementary Note-5: The screen on the substrate for a long duration

The situation where the screen is made to stay on the substrate for a long time where the deposited formulation evaporated with the screen still on the substrate is shown in **Figure** 5, as can be seen clearly, there exists a regular pattern of the material deposited, which results due to the accumulation of the deposited materials in the gaps where each thread overlaps with each other.

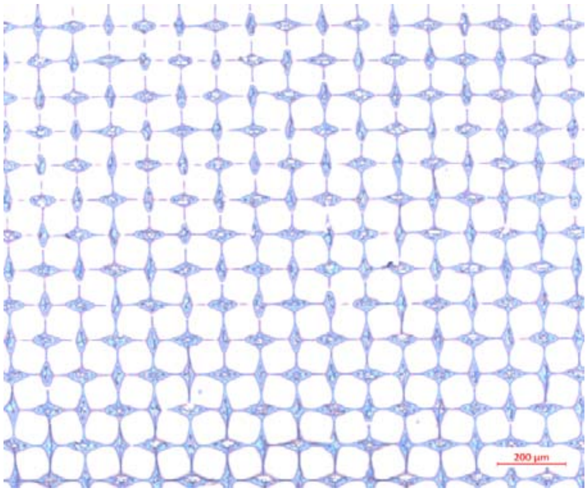

Figure 5. Regular patterns of the material of interest upon leaving the mesh for a minute

Supplementary Note- 6-8: Thickness measured by using AFM

Figure 6,**7** and **8** shows some of the examples of the measured thickness by AFM. The trench is made by scratching the substrate with a needle[7]. Then the thickness is measured along the trench by using tapping mode AFM. Then a profiling is performed over the scratch 10times, the position of the scratches are shown in the **Figure 6-8.** Then with the aid of the average of the 10 profiles we estimated the thickness of the deposited thin film**.** The 3D topography image taken by the AFM along with the measured correctional average thickness profile helps us visualize the estimation of the thickness measured. The average of the 10 profiles in the figure are used to estimate the thickness.

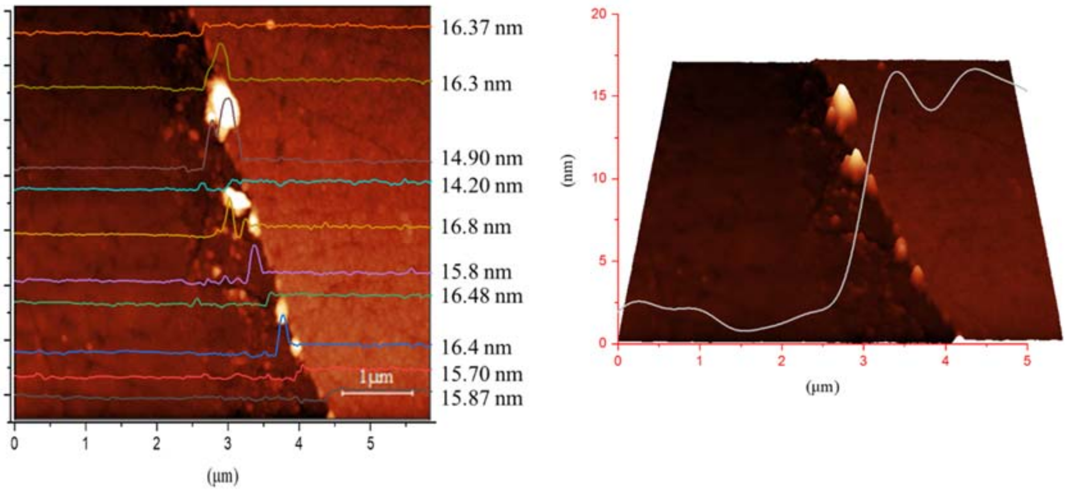

Figure 6. Example of the thickness measured from the AFM on a PEDOT:PSS coated sample showing the average thickness of 15.8 nm

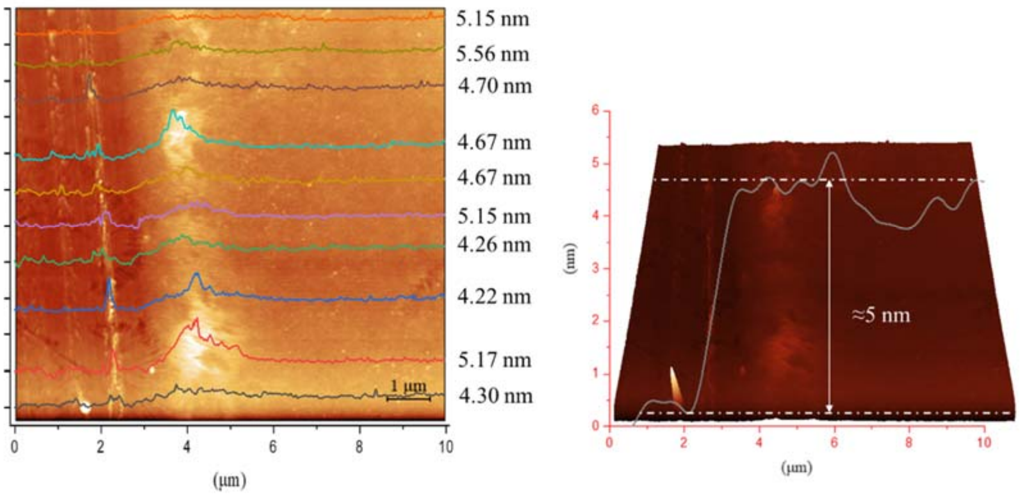

Figure 7. Example of the thickness measured from the AFM on a PEIE coated sample showing the average thickness of 4.8 nm.

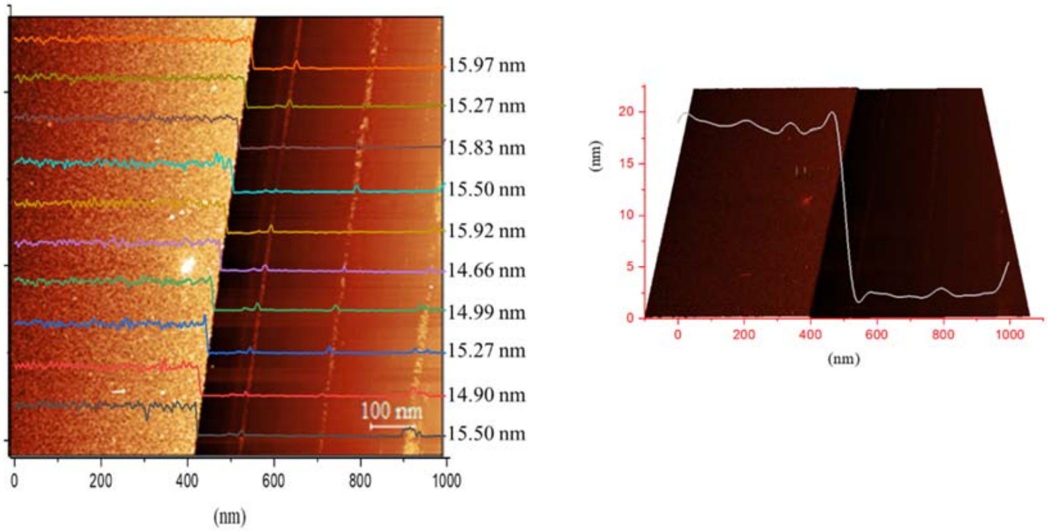

Figure 8. Example of the thickness measured from the AFM on a ZnO + PEIE coated sample showing the average thickness of 15.3 nm.

Supplementary Note-9 and 10:Thickness and surface roughness measured on large 5×5 cm^2 ITO substrate

In order to establish the versatility of the spray on screen, the large ITO substrate with area of 25cm^2 is considered for depositing the PEIE polymer. And the results show that, thickness of 5.9 nm is achieved which is as same as for the small substrate, which shows the upscaling capabilities of the spray on screen. Also the surface roughness is 0.5 nm, which is also similar to the spin coated as well as the small spray on screen coated sample.

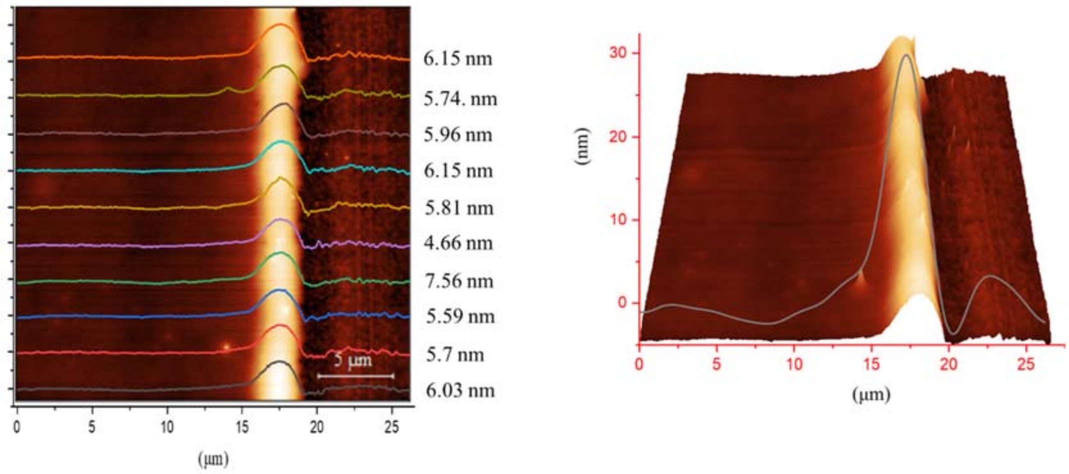

Figure 9. Thickness measurement for the PEIE film over large 5×5 cm^2 ITO substrate. The obtained thickness of 5.93nm is almost as same as shows the upscalability of the spray-on-screen.

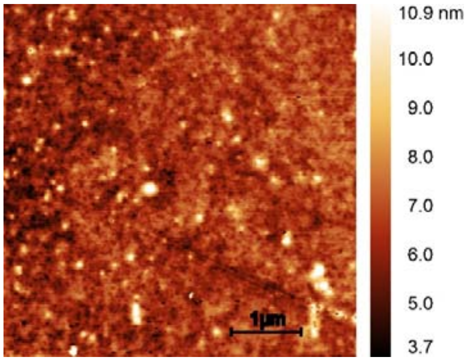

Figure 10. The surface roughness for the PEIE film over a large 5×5 cm^2 ITO with a surface roughness of 0.5nm

Supplementary Note-11 and 12: Elemental composition of the PEDOT: PSS and PEIE-ZnO nanocomposite deposited on ITO coated glass substrate.

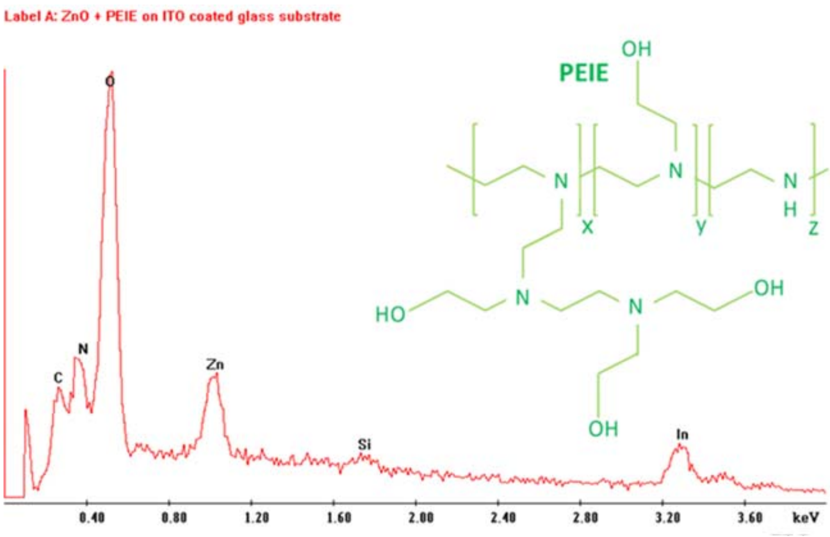

Figure 11. EDX on the PEIE-ZNO nanocomposite layers on the ITO-Coated glass substrates.

The above EDX scan is performed at 5.0 kV. For the EDX measurements ITO coated glass substrates were used, over this substrate, PEIE-ZnO nanocomposite layers were coated by spray on screen. PEIE has nitrogen. The EDX results show the presence of nitrogen and zinc. EDX also show the presence of Indium and silicon, it is because of the base substrate.

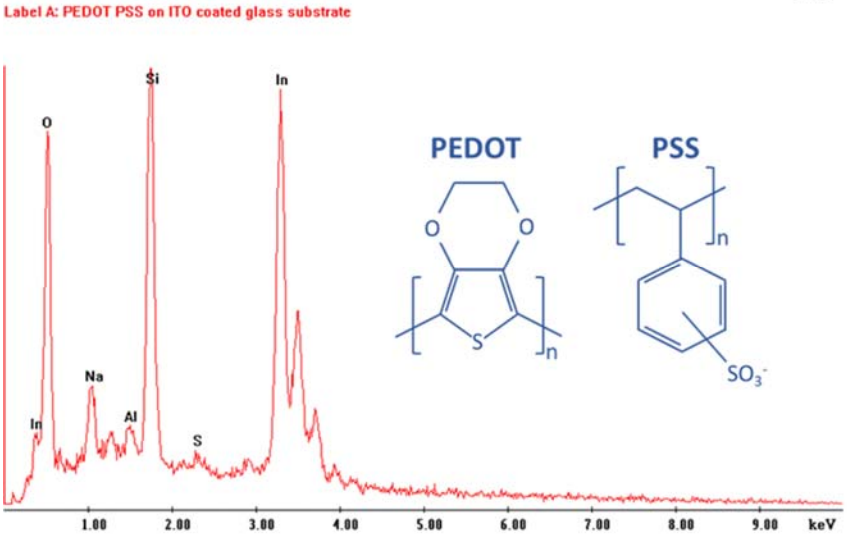

Figure 12. EDX on the PEDOT:PSS layers on the ITO-Coated glass substrates.

The above EDX scan is performed at 10 kV. For the EDX measurements, ITO coated glass substrates were used, over this substrate, PEDOT:PSS layers were coated by spray on screen. The EDX shows the presence of sulphur which is a dopant in the organic semiconductor. Since the scan was performed at 10 kV, the contribution of components which makes the composition of ITO-coated glass substrate is also visible. The borosilicate glass has the contribution from sodium and aluminium along with boron and silicon.

Supplementary Note-13-16: AFM images for the deposited coating

Figure 13, **14** and **15**, shows AFM images for the samples consisting of PEDOT:PSS, PEIE and ZnO-PEIE composite on the substrate of interest. These samples were fabricated by using spin coating as the deposition technique. These are measured as a reference for spray on screen technology.

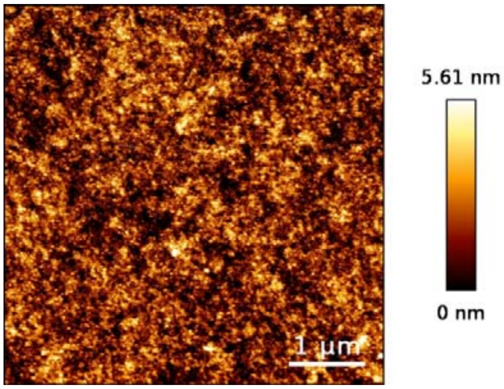

Figure 13. The topographical image of the PEDOT:PSS deposited by spin coating showing average surface roughness of 1.008 nm

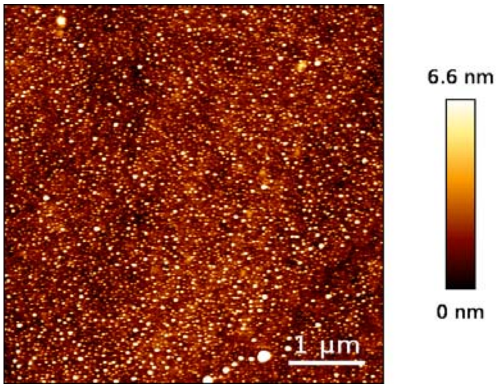

Figure 14. The topographical image of the PEIE deposited by spin coating showing average surface roughness of 0.8 nm

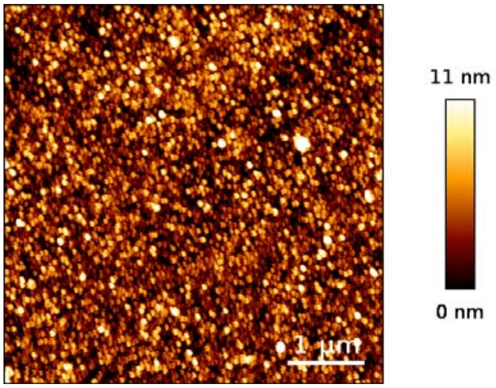

Figure 15. The topographical image of the PEIE-ZnO deposited by spin coating showing average surface roughness of 1.9 nm

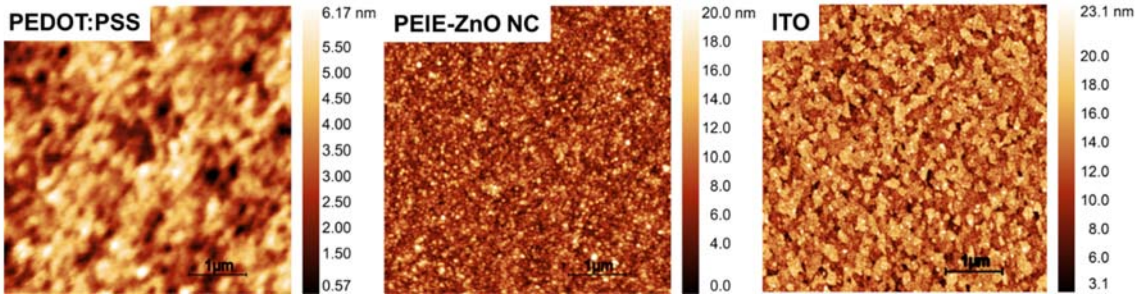

Figure 16. Surface roughness comparison of the spray on screen deposited PEDOT:PSS and PEIE-ZnO nanocomposite in comparison with the bare ITO substrate. Surface roughness: ITO- 3.21 nm, PEDOT:PSS, 1nm and PEIE-ZnO nanocomposite- 2.69 nm

Supplementary Table 1. Thickness variation by Spray-on-Screen for different materials

The versatility of the Spray-on-Screen to tune the thickness of the deposited film by using the optimized parameters of the USSC and the screen mesh, but varying the solution concentration, is demonstrated in **Table 1** for different materials.

Table 1. Thickness variation for different material for varying concentration

Material	0.01 ml PEIE/35 ml IPA	0.025 ml PEIE/35 ml IPA	0.05 ml PEIE/35 ml IPA	0.07 ml PEIE/35 ml IPA	0.1 ml PEIE/35 ml IPA
PEIE	4.8 nm	10.08 nm	20.1 nm	25.6 nm	38.2 nm

Material	1 ml PEDOT:PSS/30 ml water	1 ml PEDOT:PSS/20 ml water	1 ml PEDOT:PSS/15 ml water	1 ml PEDOT:PSS/10 ml water	1 ml PEDOT:PSS/5 ml water
PEDOT:PSS	15.8 nm	22.6 nm	31.9 nm	38.2 nm	48.9 nm

Material	2ml ZnO+0.01 ml PEIE/30 ml IPA	2ml ZnO+0.01 ml PEIE/20 ml IPA	2ml ZnO+0.01 ml PEIE/15 ml IPA	2ml ZnO+0.01 ml PEIE/10 ml IPA	2ml ZnO+0.01 ml PEIE/5 ml IPA
ZnO+PEIE	15.3 nm	21.07 nm	33.7 nm	42.5 nm	57.7 nm

Supplementary Note-17: Surface energy calculation

For the surface energy measurements by using equilibrium contact angle we have used Fowkes theory[8]. Fowkes theory incorporates the equilibrium contact angle of polar and disperses solutions to determine the overall surface free energy of the substrate Super yellow and ITO substrate. In our experiments, we have used water as a polar component and diiodomethane as a dispersive component. Equilibrium contact angle measurements show a water contact angle of 90 deg and diiodomethane contact angle of 44 deg for super yellow and water contact angle of 10 deg and a diiodomethane contact angle of 26.3 deg for ITO substrates.

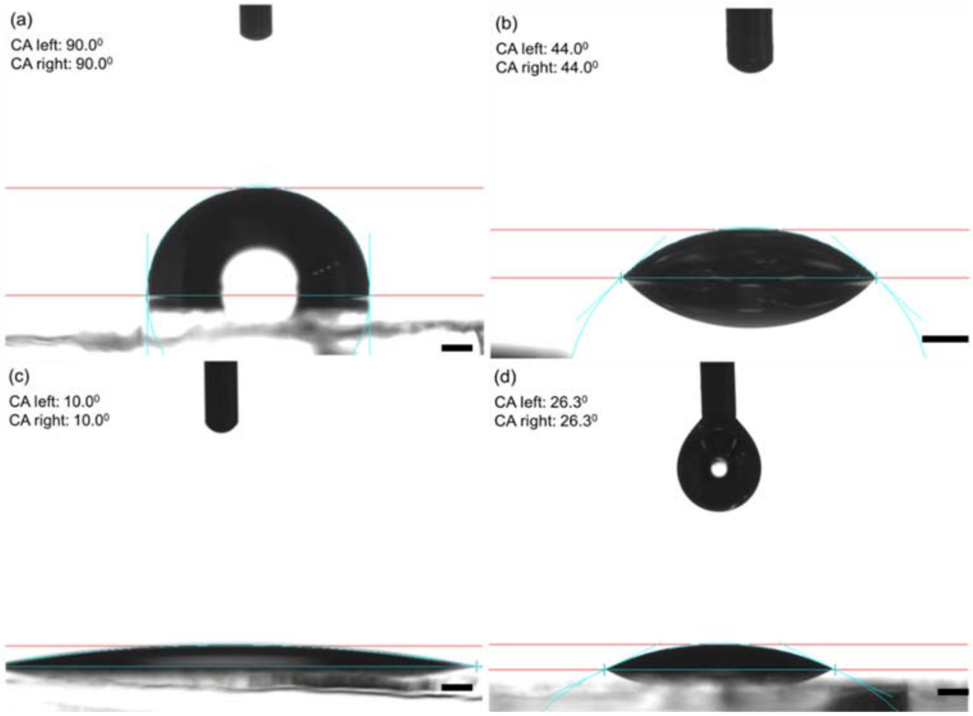

Figure 17. Contact angle measurements by using (a)(c) Water (polar component) with a contact angle of 90° on SY and the surface tension of 72.8 mN m^{-1}, contact angle of 10° on ITO substrate and (b) (d) Diiodomethane (dispersive component) with a contact angle of 44° on SY and the surface tension value of 50.8 mN m^{-1}, contact angle of 26.3° on ITO substrate (Scale 0.5 mm).

By using the relation given by Fowke's,

$$\gamma\ (dispersive) = \frac{\gamma_{diiodomethane}(cos\theta + 1)^2}{4}$$

$$\gamma\ (polar) = \frac{\gamma_{water}(cos\theta + 1)^2}{4}$$

$$\gamma\ (solid) = \gamma\ (dispersive) + \gamma\ (polar)$$

where γ is the surface tension. The total surface free energy according to Fowke's theory was found to be 56 mN m^{-1}. Similarly, the surface energy of the ITO is calculated and it was found to be around 117.37 mN m^{-1}.

Supplementary Note-18,19 and 20:Youngs modulus calculation on the super yellow substrate

An Au coated tip is used with a tip calibration was done using a clean ultra-flat gold substrate On each region of 10 μm², a grid of 100 points was set and force curves were taken at each of those points applying 2 nN. Young's modulus was calculated using the data processing software of the AFM applying the Hertz/Snedon model. All measurements were carried out in contact mode in air. The sample for the force spectroscopy was prepared by using a glass substrate following the cleaning protocol and Super yellow (SY) solution is spin coated on the glass substrate. The SY is rotated at 750 rpm for 60 seconds.

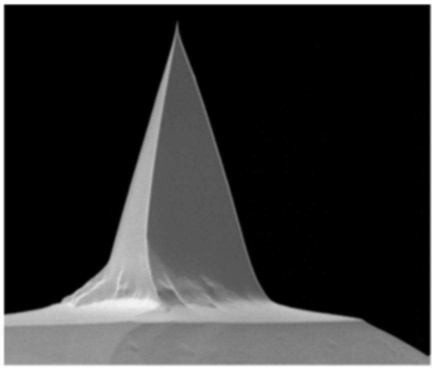

Figure 18. Triangular Au coated tip used in the experiments

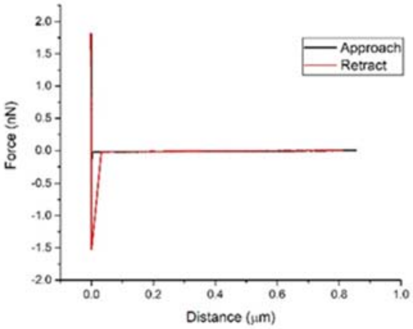

Figure 19. Calibration curve of the Au-tip

(A) (B)

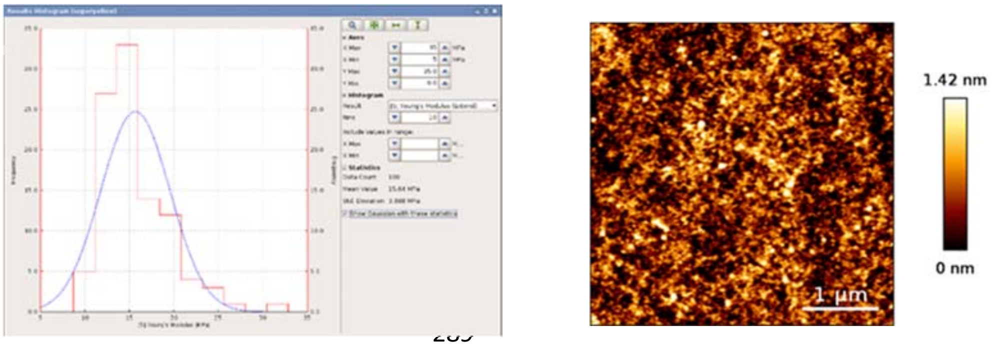

Figure 20. Calculated Young's modulus of the super yellow substrate using the data processing software of the AFM applying the Hertz/Snedon model and the surface topography of the scanned region.

Supplementary Note-21: OLEDs construction

The architecture chosen for the present study involves, ITO (135nm) as an anode, PEDOT:PSS (30nm) as an electron injection layer, super yellow (80nm) as an active layer, ZnO-PEIE nanocomposite (~16nm) or calcium (30nm) as an electron injection layer and finally aluminium (80nm) as a cathode. The prepatterned ITO substrates are thoroughly cleaned at first by ultrasonication and later with UV-Ozone. The ultrasonication involves 30 min in soap solution, 20 min in demineralized water, 10 min in acetone and 10 min in isopropanol alcohol. The ultrasonicated samples are dried by nitrogen gas for 1 min. Later these samples were transferred into a UV-ozone system. For the state-of the-art standard devices, the PEDOT:PSS is spin coated outside the glovebox and transferred into the glovebox where SY is spin coated. For calcium based devices, the samples were transferred into the evaporation chamber where calcium and aluminium are evaporated. For spin coated standard devices, the ZnO-PEIE nanocomposite is spin coated and transferred into the evaporation chamber to deposite aluminium. For spray-on-screen devices, at first PEDOT:PSS is SoS deposited outside the glovebox followed by SY deposition inside the glovebox and later ZnO-PEIE nanocomposite layer is deposited by SoS outside the glovebox. Finally, the devices were transferred into evaporation chamber for the aluminium deposition. All the samples are characterized inside the glovebox. Where the combination of Keithly power source and integrating sphere is used for JVL measurements. An home built silicon photodetector is used for EQE measurements.

Supplementary References:

1. Gavim, A. E. X. *et al.* Water-suspended MoO3 nanoparticles prepared by LASIS and fast processing as thin film by ultrasonic spray deposition. *Sol. Energy Mater. Sol. Cells* **200**, (2019).

2. Engineering, U. S. & Weldon, P. E. United States Patent (19). (1996).

3. Stephan, I., Bush, G. & Stahley, R. E. United States Patent (19). (2000).

4. Erickson, S. J. *et al.* (12) Patent Application Publication (10) Pub. No.: US 2008/0314314 A1. **1**, (2008).

5. Verding, P., Deferme, W. & Steffen, W. Velocity and size measurement of droplets from an ultrasonic spray coater using photon correlation spectroscopy and turbidimetry. *Appl. Opt.* **59**, 7496 (2020).

6. Xuefeng, G. & Lei, J. Water-repellent legs of water striders. *Nature* **432**, 2004–2004 (2004).

7. Ton-That, C., Shard, A. G. & Bradley, R. H. Thickness of spin-cast polymer thin films determined by angle-resolved XPS and AFM tip-scratch methods. *Langmuir* **16**, 2281–2284 (2000).

8. Fowkes, F. M. Attractive forces at interfaces. *Ind. Eng. Chem.* **56**, 40–52 (1964).
