# Supplementary material for: Deposition of ultra-thin coatings by a nature-inspired Spray-on-Screen technology
Source: Commun Eng. 2023 Jun 22;2:42. doi: 10.1038/s44172-023-00093-0 (PMC10955976; doi:10.1038/s44172-023-00093-0)
Supplement: Supplementary file 3 — Description of Additional Supplementary Files [file 44172_2023_93_MOESM3_ESM.pdf]

Description of Additional Supplementary Files

File name: Supplementary Video 1

Description: Supplementary video 1 shows the formation of microdroplets in the range of <100 µm with the USSC. To capture the slow-motion video of the droplets generated, A high-speed camera, Photron Mini AX100 200 K-M- 32 GB, is mounted together with a bi-telecentric objective, Thorlabs, X2, MVTC23200, as a reference measurement system is used. Illumination of the view field is done with a white light source, SCHOTT, KL 2500 LCD, in combination with a telecentric back-light illuminator, Techspec, 52 mm. Since the velocity of the droplets was high, to capture a precise video, a frame rate of 20,000 frames/second was selected. However, this is done at the expense of a reduction in the field of view for the camera to attain this frame rate. Therefore, in the present experiment, the resolution for a 20 µm particle is almost equal to 4 pixels. The video is slowed down by 2000 times. The printing parameters for the video captured are Shroud pressure: 1 psi, flow rate of 1.5 ml min-1, nozzle power of 2.5 W, the solvent being used is water.

File name: Supplementary Video 2

Description: The supplementary video 2 shows the working principle of SoS technology, where the microdroplets are generated in the nozzle head of USSC, and these microdroplets are made to deposited on the screen mesh. the microdroplets are converted into milli droplets on the screen mesh. This is followed by applying external pressure on the screen where due to capillary force, the liquid is drawn onto the substrate underneath the screen mesh.

File name: Supplementary Video 3

Description: The supplementary video 3 shows the small area and large area OLEDs built by SoS. The OLEDs were lit up by passing the current. The light illumination as well as change in intensity of the light output with respect to the applied potential could be seen. The uniformity of the deposition of the EIL layer on the highly sensitive SY layer over a large area provides a new avenue for the SoS to be used for other application where ultrathin films are crucial.
